# GC/MS-based metabolomic analysis of cerebrospinal fluid (CSF) from glioma patients

**DOI:** 10.1007/s11060-013-1090-x

**Published:** 2013-03-01

**Authors:** Satoshi Nakamizo, Takashi Sasayama, Masakazu Shinohara, Yasuhiro Irino, Shin Nishiumi, Masamitsu Nishihara, Hirotomo Tanaka, Kazuhiro Tanaka, Katsu Mizukawa, Tomoo Itoh, Masaaki Taniguchi, Kohkichi Hosoda, Masaru Yoshida, Eiji Kohmura

**Affiliations:** 1 Department of Neurosurgery, Kobe University Graduate School of Medicine, 7-5-1, Kusunoki-cho, Chuo-ku, Kobe, 650-0017 Japan; 2 Division of Gastroenterology, Kobe University Graduate School of Medicine, Kobe, Japan; 3 Division of Lipid Biochemistry, Kobe University Graduate School of Medicine, Kobe, Japan; 4 Division of Metabolomics Research, Kobe University Graduate School of Medicine, Kobe, Japan; 5 The Integrated Center for Mass Spectrometry, Kobe University Graduate School of Medicine, Kobe, Japan; 6 Division of Pathology, Kobe University Graduate School of Medicine, Kobe, Japan; 7 Department of Neurosurgery, West Kobe Medical Center, Kobe, Japan

**Keywords:** Metabolome, Glioma, Cerebrospinal fluid, Lactic acid, Citric acid, Isocitrate dehydrogenase

## Abstract

Metabolomics has recently undergone rapid development; however, metabolomic analysis in cerebrospinal fluid (CSF) is not a common practice. We analyzed the metabolite profiles of preoperative CSF samples from 32 patients with histologically confirmed glioma using gas chromatography/mass spectrometry (GC/MS). We assessed how alterations in the metabolite levels were related to the World Health Organization (WHO) tumor grades, tumor location, gadolinium enhancement on magnetic resonance imaging (MRI), and the *isocitrate dehydrogenase* (*IDH*) mutation status. Sixty-one metabolites were identified in the CSF from glioma patients using targeted, quantitative and non-targeted, semi-quantitative analysis. The citric and isocitric acid levels were significantly higher in the glioblastoma (GBM) samples than in the grades I–II and grade III glioma samples. In addition, the lactic and 2-aminopimelic acid levels were relatively higher in the GBM samples than in the grades I–II glioma samples. The CSF levels of the citric, isocitric, and lactic acids were significantly higher in grade I–III gliomas with mutant *IDH* than in those with wild-type *IDH*. The tumor location and enhancement obtained using MRI did not significantly affect the metabolite profiles. Higher CSF levels of lactic acid were statistically associated with a poorer prognosis in grades III–IV malignant gliomas. Our study suggests that the metabolomic analysis of CSF from glioma patients may be useful for predicting the glioma grade, metabolic state, and prognosis of gliomas.

**Electronic supplementary material:**

The online version of this article (doi:10.1007/s11060-013-1090-x) contains supplementary material, which is available to authorized users.

## Introduction

Cerebrospinal fluid (CSF) has various functions, such as protecting the brain, transporting biological substances, and excreting toxic and waste substances. CSF is in direct contact with the extracellular fluid of the brain. Although the CSF composition reflects that of the blood plasma, active transport from the blood and secretions from the brain contribute to the CSF composition. Therefore, the CSF composition can indicate biological brain processes, and CSF analysis is indispensable for diagnosing and understanding central nervous system (CNS) disorders [1–4]. A chemical examination of CSF is an important tool for the diagnosis of some types of brain tumors [5, 6].

Gliomas, the most common primary CNS tumors, are classified as grades I to IV based on the histopathological and clinical criteria established by the WHO [7]. Grade I gliomas, which are considered benign, are generally curable with complete surgical resection [8]. In contrast, Grades II and III gliomas are invasive, progress to higher-grade lesions, and have poor clinical outcomes. Grade IV gliomas are glioblastomas (GBMs), which are the most invasive and have a dismal prognosis [9, 10]. Metabolic remodeling is a predominant phenotype of malignant tumor cells and refers to the alteration of the utilization and/or synthesis of important metabolites, including glucose, fatty acids, and amino acids, by tumor cells [11]. The levels of several metabolites, such as lactic acid and choline, are elevated in malignant gliomas [12]. Recently, mutations in the *isocitrate dehydrogenase* (*IDH*) *genes* have been identified in gliomas [13, 14]. Both IDH1 and IDH2 are NADP^+^-dependent dehydrogenases and convert isocitrate into α-ketoglutarate. Previous reports have indicated that these mutations are frequently observed in astrocytic and oligodendroglial tumors of grades II and III [14–17]. Because these enzymes catalyze reactions of energy metabolism, *IDH* mutations may alter global cellular metabolism [18].

Metabolomics has recently undergone rapid development. Metabolomics includes the analysis of metabolites from biofluids or tissues using nuclear magnetic resonance (NMR) or mass spectrometry (MS)-based approaches, including liquid chromatography/mass spectrometry (LC/MS) or gas chromatography/mass spectrometry (GC/MS). To date, the global metabolic profiling of human biofluids, such as urine and sera, has been used to visualize the distinct metabolic profiles of patients with cancer and gastroenterological disease [19]. Furthermore, the metabolic profiling of tissue specimens from several cancer patients has revealed significant variations in the metabolites detected in tumors versus normal tissue [20]. There have also been a small number of reports on the metabolomic analysis of CSF in CNS disorders [21–26]. However, few metabolomic studies using MS-based methods have been performed on the CSF of glioma patients. In this study, we conducted a GC/MS-based metabolomic analysis of CSF samples from 32 glioma patients. We examined the differences in the metabolites of the CSF samples using various clinical parameters, such as WHO grades and *IDH* mutation. Our study indicates that a metabolomic analysis of CSF from glioma patients may be useful for predicting the malignancy grade and *IDH* mutation status.

## Materials and methods

### Subjects

Our study included 32 patients with intracranial glial tumors, and the information concerning the participants is summarized in Table 1. Patients with metabolism-related disorders, such as hypertension, diabetes, and hyperlipidemia, were excluded from this study. The patients were treated in the Department of Neurosurgery, University of Kobe, between January 2008 and November 2010. For the preoperative diagnosis, 2–5 ml of CSF was drawn from the patients using a lumbar puncture, when not contraindicated, after receiving informed consent. Pathological diagnoses were obtained by either performing brain biopsies or removing the tumors. The tumor pathology was centrally reviewed by a neuropathologist. This study was approved by the ethics committee of the Kobe University Graduate School of Medicine.

**Table 1:** Patient characteristics

No	Sex	Age	Pathology	WHO grade	Tumor location	Tumor size (cm^3^)	Proximal to ventricle	Gd-enhance	IDH1 mutation	IDH2 mutation	Prot (mg/dl)	Glu (mg/dl)	LDH (IU/l)
1	F	37	PA	Grade I	Cerebellum	6.0	+	+	−	−	27	84	18
2	M	43	PA	Grade I	Medulla	3.0	−	+	+	−	−	−	−
3	M	28	DA	Grade II	Basal ganglia	12.8	−	−	−	−	37	64	16
4	M	66	DA	Grade II	Fronto-temporal	64.0	+	−	−	−	34	63	34
5	F	75	DA	Grade II	Temporal	46.0	+	−	−	+	−	−	−
6	M	35	OG	Grade II	Frontal	13.9	−	+	+	−	31	63	16
7	F	60	OG	Grade II	Frontal	52.6	−	−	+	−	26	71	25
8	M	64	OG	Grade II	Frontal	48.2	−	−	+	−	40	63	26
9	F	16	EP	Grade II	Third ventricle	7.0	+	+	−	−	−	−	−
10	M	25	EP	Grade II	Lateral ventricle	3.7	+	+	−	−	24	71	−
11	F	18	AA	Grade III	Thalamus	28.6	+	+	−	−	29	63	−
12	F	31	AA	Grade III	Temporal	35.8	−	−	−	−	−	−	−
13	M	36	AA	Grade III	Thalamus	23.9	−	+	−	−	6	81	<10
14	M	45	AA	Grade III	Temporo-parietal	7.7	+	+	−	−	56	60	29
15	F	66	AA	Grade III	Frontal	6.7	−	−	−	−	46	67	29
16	M	35	AOG	Grade III	Frontal	104.3	+	+	+	−	25	55	12
17	F	59	AOG	Grade III	Temporal	22.0	−	−	−	−	48	69	21
18	F	24	AEP	Grade III	Parietal	67.7	−	+	−	−	53	53	11
19	F	39	GBM	Grade IV	Callosum	88.8	+	+	−	−	56	70	14
20	M	41	GBM	Grade IV	Temporal	61.2	+	+	−	−	25	64	17
21	F	54	GBM	Grade IV	Callosum	45.4	+	+	−	−	46	71	−
22	F	56	GBM	Grade IV	Temporal	25.6	−	+	−	−	71	69	18
23	F	57	GBM	Grade IV	Callosum	20.8	+	+	−	−	186	51	27
24	M	60	GBM	Grade IV	Frontal	15.0	+	+	−	−	41	116	−
25	M	60	GBM	Grade IV	Parietal	74.5	+	+	−	−	145	75	93
26	M	64	GBM	Grade IV	Frontal	20.8	−	+	−	−	102	78	15
27	F	65	GBM	Grade IV	Cerebellum	19.3	+	+	−	−	169	49	36
28	M	68	GBM	Grade IV	Optic nerve	2.1	−	+	−	−	76	120	19
29	M	68	GBM	Grade IV	Fronto-temporal	21.9	−	+	−	−	87	68	20
30	M	74	GBM	Grade IV	Frontal	26.5	+	+	−	−	67	67	35
31	M	79	GBM	Grade IV	Temporal	6.9	−	+	−	−	64	62	46
32	F	79	GBM	Grade IV	Temporal	81.4	+	+	−	−	125	82	47

*PA* pilocytic astrocytoma, *DA* diffuse astrocytoma, *OG* oligodendroglioma, *AA* anaplastic astrocytoma, *AOG* anaplastic oligodendroglioma, *AEP* anaplastic ependymoma, *GBM* glioblastoma. *Prot* protein, *Glu* glucose, *LDH* lactate dehydrogenase

### CSF collection and preparation

The CSF samples were centrifuged, and the supernatants were transferred to fresh tubes and stored at −80 °C until use. Fifty microliters of CSF were mixed with 250 μl of a solvent mixture (MeOH:H_2_O:CHCl_3_ = 2.5:1:1) containing 10 μl of 1.0 mg/ml 2-isopropylmalic acid dissolved in distilled water as an internal standard. The solution was shaken for 30 min at 37 °C and centrifuged. A total of 250 μl of the resultant supernatant was transferred to a fresh tube, and 200 μl of distilled water was added. After mixing, the solution was centrifuged, and 250 μl of the resultant supernatant was transferred to a tube and lyophilized using a freeze dryer. For oximation, 40 μl of 20 mg/ml methoxyamine hydrochloride dissolved in pyridine was mixed with the lyophilized sample and shaken at 1,200 rpm for 90 min at 30 °C. Next, 20 μl of *N*-methyl-*N*-(trimethylsilyl)trifluoroacetamide (MSTFA) was added for the derivatization step, and the mixture was mixed at 1,200 rpm for 30 min at 37 °C. The mixture was then centrifuged, and the resultant supernatant was subjected to GC/MS measurements. To assess the technical variation in the metabolomic experiments, each of the samples was extracted, derivatized, and measured in six replicates. Then, the average of the data set per sample was calculated.

### Targeted, quantitative analysis by GCMS-QP2010 Plus

GC/MS analysis was conducted according to the previously reported modified method [27, 28]. This analysis used a DB-5 column (30 m × 0.25 mm i.d.; film thickness: 1.00 μm) (J&W Scientific, Folsom, CA, USA). The GC column temperature was programmed to increase from 100 to 320 °C at a rate of 4 °C/min, and the total GC run time was 60 min. The injection volume was 1 μl in the splitless mode, and the mass conditions were set as follows: ionization voltage, 70 eV; ion source temperature, 200 °C; and full scan mode, range of 35–600 *m*/*z* and scan velocity 0.20 scans/second.

### Non-targeted, semi-quantitative analysis using GCMS-QP2010 Ultra

The GC/MS analysis was conducted according to the method described by Tsugawa et al. [29]. This analysis used a fused silica capillary column (CP-SIL 8 CB low bleed/MS; 30 m × 0.25 mm inner diameter, 0.25 μm film thickness; Agilent Co., Palo Alto, CA). The column temperature was maintained at 80 °C for two minutes isothermally, then increased by 15 °C/min to 330 °C, and maintained for 6 min isothermally. The injection volume was 1 μl. The transfer line and ion-source temperatures were 250 and 200 °C, respectively. In brief, 20 scans/second were recorded over the mass range 85–500 *m*/*z* by using the Advanced Scanning Speed Protocol (ASSP, Shimadzu Co.). The peak detection and alignment were performed using MetAlign software (Wageningen UR, The Netherlands). The resulting data were analyzed using in-house analytical software (AI output).

### Multiple classification analysis (multivariate analysis)

We constructed a three-dimensional matrix using the sample names (observations), metabolite numbers (variable indices), and normalized peak intensities (variables). A principal component analysis (PCA) was performed using commercially available SIMCA-P + Software version 12.0.1 (Umetrics, Umeå, Sweden).

### Mutation analysis of IDH1 and IDH2

Genomic DNA was isolated from paraffin blocks of glioma tissue using the DNeasy FFPE kit (Qiagen, Valencia, CA, USA), according to the manufacturer’s instructions. To detect the *IDH* mutations, forward and reverse primers were designed to amplify exon 4 (codon R132) of the *IDH1* gene and exon 4 (codon R172) of the *IDH2* gene. The polymerase chain reaction products were sequenced using the primers and the BigDye Terminator v3.1 Cycle Sequencing kit (Applied Biosystems, Inc, Foster City, CA, USA).

### Statistical analysis

The statistical significance between two groups was determined using the Mann–Whitney *U* test. The statistical significance among three groups was determined using the Steel–Dwass test. Multiple tests were controlled by false discovery rate (FDR), the expected proportion of false positive results that is declared significant [30]. Q < 0.15 was considered to be statistically significant. Survival was estimated using the Kaplan–Meier method, and significance was determined using the log-rank test. *P* < 0.05 was considered to be statistically significant. We divided the patients into two groups: the “higher (larger) group” had levels higher than the median, and the “lower (smaller) group” had levels lower than the median. Statistical analysis was performed using the SPSS, version 12.0, software package. FDR analysis was performed using the R, Q-value package (version 2.15.2) (R Foundation for Statistical Computing, Vienna, Austria).

## Results

### Metabolite profiling of the CSF from glioma patients and the association with glioma malignancy

**Fig. 1:**
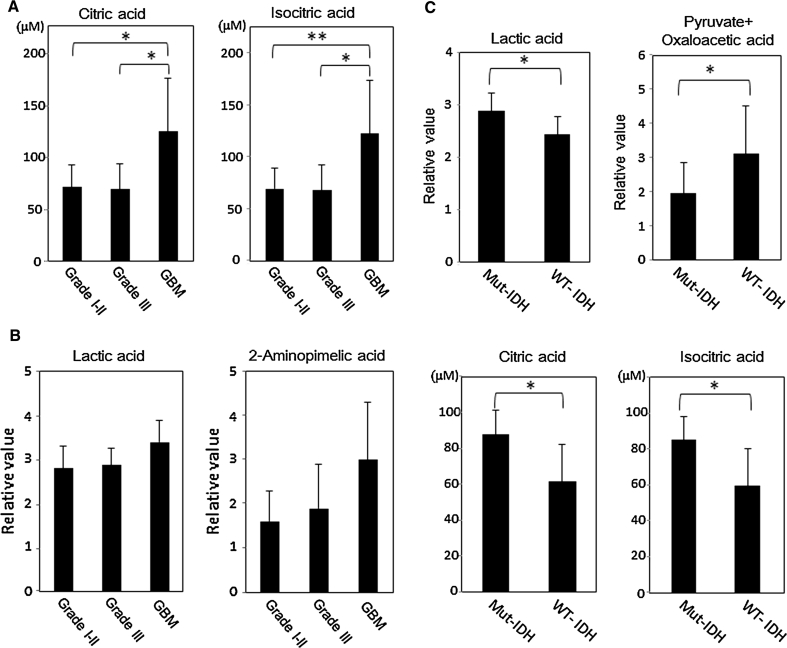
**a**, **b** Quantitative level (**a**) and Semi-quantitative level (**b**) of metabolites that exhibit significant differences among the grades I–II gliomas, grade III gliomas, and GBMs. The *columns* are the average of each group; bars, SD. *p* values were calculated using the Steel–Dwass test (*p < 0.05, **p < 0.01). **c** Quantitative and semi-quantitative metabolite levels that exhibit significant differences between the mutant *IDH* group versus the wild-type *IDH* group in grade I–III gliomas except for GBMs. The *columns* are the average of each group; *bars*, SD. *p* values were calculated using the Mann–Whitney nonparametric *U* test (*p < 0.05, **p < 0.01)

CSF samples from 32 glioma patients were subjected to metabolite level measurements with GC/MS (Table 1). A total of 16 metabolites involved in the TCA cycle, glycolysis, or amino acids were quantified (Table 2). The fold induction of the peak height value was calculated and compared among the three groups (grades I–II, grade III, and GBM). The results are listed in Supplementary Table S1. There was no significant difference in metabolite levels between grades I–II and grade III gliomas. In contrast, the levels of the following two molecules were significantly increased in the GBMs compared with grades I–II or III gliomas: citric acid (1.77-fold, vs. grades I–II (*p* = 0.0125, Q = 0.1002); 1.83-fold, vs. grade III (*p* = 0.0174, Q = 0.0552)) and isocitric acid (1.78-fold, vs. grades I–II (*p* = 0.0096, Q = 0.1002); 1.83-fold, vs. grade III (*p* < 0.0174, Q = 0.0552)) (Fig. 1a). In addition, the citric and isocitric acid levels were significantly increased in GBMs compared with the levels observed in anaplastic astrocytomas (citric acid: *p* = 0.033, Q = 0.039, isocitric acid: *p* = 0.033, Q = 0.039), and fumaric acid was significantly decreased in GBMs compared with anaplastic astrocytomas (*p* = 0.033, Q = 0.070). (Supplementary Fig. S2). The concentration of downstream molecules of isocitric acid in the TCA cycle, i.e., succinic acid, fumaric acid, and malic acid, were relatively lower in GBMs than in grades I-II or III gliomas (Supplementary Table S1). None of the amino acids or their derivatives manifested significant differences among each grade of gliomas.

**Table 2:** Metabolite concentrations in 32 CSF samples as measured by a GCMS-QP2010 Plus

Compound name	Grade I–II (*n* = 10)	Grade III (*n* = 8)	GBM (*n* = 14)	*p* value
	Mean ± SD (μM)	Mean ± SD (μM)	Mean ± SD (μM)	
Succinic acid	2.26 ± 1.20	3.50 ± 3.69	1.96 ± 1.03	
Fumaric acid	1.01 ± 0.859	1.67 ± 1.76	0.757 ± 0.311	
Malic acid	2.08 ± 4.14	6.12 ± 10.8	0.400 ± 0.195	
Aconitic acid	8.43 ± 3.91	7.61 ± 3.84	13.8 ± 7.45	
Isocitric acid	68.8 ± 20.0	67.1 ± 24.2	121 ± 50.4	*p* = 0.001(I–II/IV), *p* = 0.017(III/IV)
Citric acid	70.3 ± 20.8	67.8 ± 24.3	123 ± 50.1	*P* = 0.013(I–II/IV), *p* = 0.017(III/IV)
Alanine	41.5 ± 27.8	34.2 ± 5.89	43.8 ± 11.0	
Valine	19.0 ± 10.8	14.3 ± 2.97	20.2 ± 8.95	
Leucine	14.2 ± 4.63	11.7 ± 3.09	15.0 ± 5.28	
Isoleucine	7.64 ± 2.62	5.97 ± 1.80	7.56 ± 2.83	
Proline	7.25 ± 9.45	3.78 ± 1.28	4.32 ± 1.62	
Serine	26.4 ± 14.3	26.7 ± 9.76	22.2 ± 5.71	
Threonine	27.3 ± 10.0	23.6 ± 4.89	27.5 ± 6.84	
Methionine	7.30 ± 1.54	6.78 ± 1.29	7.64 ± 1.46	
Phenylalanine	11.1 ± 1.32	11.1 ± 1.25	13.2 ± 2.69	
Tyrosine	11.7 ± 2.51	11.8 ± 1.72	12.3 ± 2.02	

*SD* standard deviation

We identified 45 metabolites with a non-targeted semi-quantitative analysis that was performed using a GCMS-QP2010 Ultra (Supplementary Tab S2). The levels of lactic acid (1.21-fold, *p* = 0.033) and 2-aminopimelic acid (1.87-fold, *p* = 0.042) were higher in GBMs than in grades I–II gliomas (Fig. 1b, Supplementary Table S3). However, a high Q-value indicated that these differences were not significant (lactic acid: Q-value = 0.955, 2-aminopimelic acid: Q-value = 0.955). None of the metabolite levels was significantly different between grade III gliomas and grades I–II gliomas or GBMs. In addition, there were no significant differences between grade I gliomas and grade II gliomas. The patterns of variation in the CSF metabolites were analyzed to assess the clustering of each WHO grading group, using the multivariate statistics from principal component analysis (PCA). However, the 2D-PCA score plots of the principal components failed to demonstrate distinct clustering or a clear separation of the three groups (data not shown).

### The CSF metabolites appear to reflect the metabolic conditioning of gliomas

To determine the source of the increased levels of lactic acid, we examined the expression of LDHA (lactate dehydrogenase-A), which produces lactic acid from pyruvate, in all glioma samples analyzed. Overall, the LDHA was predominantly expressed in tumor cells rather than in vascular tissues, as shown in the Supplementary Fig. S3. Notably, the tumor cells near the necrotic tissues markedly expressed LDHA (Supplementary Fig. S3i). From these findings, tumor cells, particularly hypoxic tumor cells, appear to predominantly produce and release lactic acid. The comparison of the LDHA expression level of GBMs and other gliomas demonstrated that LDHA expression is stronger in GBMs than in other gliomas (Supplementary Fig. S3). This finding is consistent with the results demonstrated for the CSF lactic acid levels.

Because 1H-MRS studies were performed for 9 patients in this study, we compared the CSF metabolite levels measured by GC/MS with those of tumors measured by 1H-MRS. The MRI and 1H-MRS images of 3 patients are indicated in Supplementary Fig. S1 (patients 10, 19, and 21 in Table 1). Although the sample number of the 1H-MRS studies was small, the lactic acid concentrations in the CSF correlated with the level of lactic acid in the tumor using 1H-MRS (Supplementary Fig. S1). Therefore, the CSF lactic acid level appears to reflect the metabolic condition of the glioma cells. Conversely, citric and isocitric acids were not clearly detected in our MRS study. These results indicate that the CSF metabolite level appear to reflect the metabolic conditioning of the gliomas.

### Differences in the metabolites levels related to tumor location, tumor size, and gadolinium enhancement

To examine the differences in the metabolite levels related to tumor location, we divided the gliomas into two groups: the tumors located proximal to or distal from the ventricles (Table 1; Supplementary Fig. S1). None of the metabolite levels differed significantly between the two groups. In high-grade gliomas alone, however, the CSF citric and isocitric acid levels were relatively higher in gliomas proximally located to the ventricle than in those distally located to the ventricle (citric acid: *p* = 0.075, isocitric acid: *p* = 0.075). Conversely, in low-grade gliomas alone, the CSF lactic acid levels were significantly higher in the group of proximally located gliomas than in distally located gliomas (*p* = 0.028). Additionally, because gadolinium enhancement of the tumor on MRI is known to involve blood–brain barrier (BBB) disruption, we determined whether there were alterations in the metabolite levels in MRI-enhanced tumors (Table 1). However, none of the metabolite levels exhibited significant differences between the two groups. However, in high-grade gliomas alone, the CSF citric and isocitric acid levels were significantly higher in gliomas with gadolinium enhancement than in those without gadolinium enhancement (citric acid: *p* = 0.019, isocitric acid: *p* = 0.019). Next, we classified the high-grade gliomas (grades III + IV) into a larger tumor size group and a smaller tumor size group. We also compared the CSF metabolite levels between these groups; however, there were no significant differences in the metabolites between the larger group and the smaller group.

### Relationship between metabolite levels and the IDH mutation in grades I–III gliomas

*IDH* mutations are quite rare in GBMs. Therefore, we analyzed the alterations in the metabolite levels according to the *IDH* mutation status in only grades I–III gliomas, except for GBMs. None of the GBMs we analyzed had *IDH* mutations. Six glioma cases possessed a mutant *IDH1* or a mutant *IDH2*, and 12 cases had wild-type *IDH1* and *IDH2* (Table 1). The levels of the following three molecules were significantly increased in gliomas with a mutant *IDH* compared with that observed in the wild-type *IDH* gliomas: citric acid (1.43-fold, *p* = 0.0114, Q = 0.0447), isocitric acid (1.42-fold, *p* = 0.0130, Q = 0.0447), and lactic acid (1.18-fold, *p* = 0.0312, Q = 0.0717) (Table 3; Fig. 1c). In contrast, the levels of pyruvate + oxaloacetic acid was significantly decreased in gliomas with a mutant *IDH* compared to those with the wild-type *IDH* (0.62-fold, *p* = 0.0492, Q = 0.0847) (Table 3; Fig. 1c). In addition, in gliomas with a mutant *IDH*, we observed a trend towards lower succinic, fumaric, and malic acid levels, which are downstream metabolites of isocitric acid in the TCA cycle. Additionally, “pyruvate + oxaloacetic acid” represents the combined signal from both metabolites, which cannot be discriminated using the technology in this present study.

**Table 3:** A comparison of the metabolite levels involved in the TCA cycle and glycolysis between the glioma with a mutated IDH and the wild-type IDH in all of the gliomas except GBMs

Compound name	Fold induction: mutated IDH/wild-type IDH	*p* value
Pyruvate + Oxaloacetic acid	**0.62**	0.049*
Citric acid	**1.43**	0.011*
Aconitic acid	1.37	0.222
Isocitric acid	**1.42**	0.013*
Succinic acid	0.60	1.00
Fumaric acid	0.40	0.190
Malic acid	0.064	0.542
Lactic acid	**1.18**	0.031*

Pyruvate + oxaloacetic acid and lactic acid were measured by a GCMS-QP2010 Ultra. The other metabolites were measured using a GCMS-QP2010 Plus. The values are the fold induction of the peak intensity value of the group with a mutated IDH and the wild-type IDH. *p* values were calculated using the Mann–Whitney nonparametric *U*-test. (* *p* < 0.05)

### Higher CSF levels of lactic acid are associated with poor prognosis in malignant gliomas

**Fig. 2:**
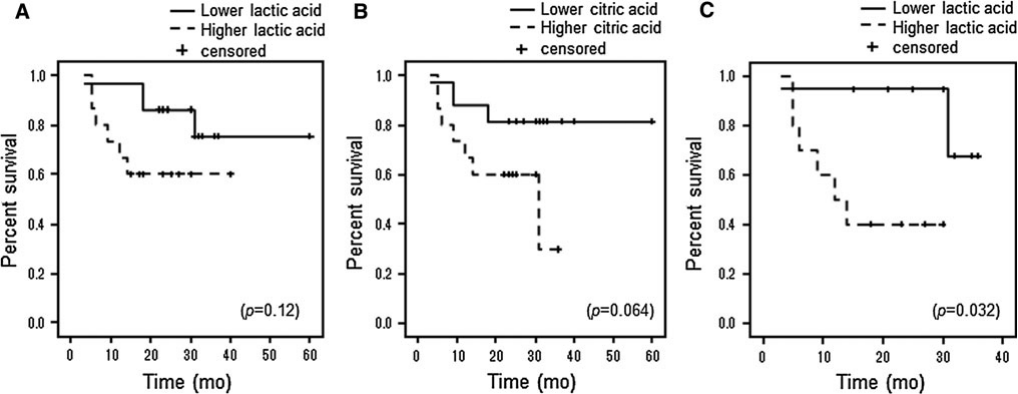
**a**, **b** In survival analysis using the Kaplan–Meier method for all glioma patients, trends were observed for higher CSF levels of lactic acid or citric acid and having shorter OS (log-rank: lactic acid; *p* = 0.12, citric acid; *p* = 0.064). **c** In malignant gliomas (grade III + IV) alone, higher CSF levels of lactic acid were significantly associated with having a shorter OS (log-rank: *p* = 0.032)

During survival analysis using the Kaplan–Meier method for all glioma patients, no significant correlation was discovered between the overall survival (OS) and the CSF level of lactic or citric acid. However, relationships were observed between higher CSF lactic or citric acid levels and having a shorter OS (log-rank: lactic acid; *p* = 0.12, citric acid; *p* = 0.064) (Fig. 2a, b). In malignant gliomas alone (grade III + IV), higher CSF levels of lactic acid were significantly associated with having a shorter OS (log-rank: *p* = 0.032) (Fig. 2c). In addition, trends were observed for higher CSF levels of citric acid and having a shorter OS (log-rank: *p* = 0.089). In grade IV (GBM) patients only, however, there was no significant correlation between the CSF lactic acid or citric acid level and OS. In multivariate analyses, however, the CSF levels of lactic acid and citric acid were not independent predictors of survival in glioma patients.

## Discussion

Metabolic remodeling is a predominant phenotype of malignant tumor cells (Warburg effect) and refers to the alteration of the utilization and/or synthesis of important metabolites, including glucose, fatty acids, and amino acids, by tumor cells [31]. Aerobic glycolysis involves the generation of substrates such as fatty acids and nucleotides that are required for rapidly proliferating cells and is associated with a survival advantage. The 13C-nuclear MR spectroscopy measurements have demonstrated that glioblastoma cells convert as much as 90 % of the glucose that they acquire into lactic acid in vitro [32]. Clinically, it is known that the levels of several metabolites, such as choline and lactic acid, are elevated in malignant glioma tissue compared with contralateral normal brain tissue. Additionally, lactic acid levels are higher in grades III and IV gliomas than in low-grade gliomas, and lipids are significantly elevated in grade IV gliomas [33]. Additionally, Colavolpe reported that malignant glioma cells absorb a large quantity of F18-2-fluoro-2-deoxy-d-glucose (FDG) and that pre-treatment with FDG-PET provides significant additional prognostic information for high-grade gliomas [34]. Thus, glioma cells have different metabolic patterns in terms of utilizing important metabolites such as glucose and fatty acids when compared with normal glial cells or among different WHO grades.

In the present study, we identified 61 metabolites in the CSF from glioma patients using GC/MS. Previous reports detected ~40–90 metabolites using GC/MS in normal human CSF [35–37], and the number of metabolites identified in this study was similar to that in the previous reports. The lactic acid level in the CSF was significantly elevated in the GBMs compared with the grades I–II gliomas. Lactic acid is often more prominent in the highest grade of glioma in MRS studies [38]. Furthermore, Yamasaki et al. [12] reported that lactic acid expression in glioma statistically correlated with a shorter OS among the MRS parameters. Consistent with their results, the higher CSF level of lactic acid tended to be associated with a shorter OS in all glioma patients and was statistically associated with a shorter OS in malignant glioma alone in our metabolomic study. Interestingly, the present study demonstrated that the CSF levels of lactic acid were significantly increased in gliomas with a mutant *IDH* compared with wild-type *IDH* in only low-grade gliomas (grades I–III). We sought to determine whether the higher lactic acid levels were associated with a poor outcome in grade I–III gliomas. However, this could not be determined because most of the patients were still alive at the end of the study. Although gliomas with a mutant *IDH* are known to be associated with improved survival, further studies are required to determine the association between the CSF lactic acid levels and the prognosis in low-grade gliomas. Lactic acid is produced by lactate dehydrogenase (LDH) and is usually an anaerobic metabolic product that occurs when the oxygen demand of a rapidly growing tumor exceeds what its neovasculature supplies. In addition, unlike most normal tissues, malignant tumor cells convert most glucose into lactic acid, regardless of whether oxygen is available to support mitochondrial oxidative phosphorylation [11]. Active glycolysis increases the cytosolic NADH/NAD^+^ ratio and thereby accelerates lactate dehydrogenase activity [39]. In the present study, LDHA was predominantly expressed in tumor cells rather than in vascular tissues. In particular, the tumor cells near the necrotic tissues markedly expressed LDHA (Supplementary Fig. 3i). From these findings, tumor cells, particularly hypoxic tumor cells, seem to predominantly produce and release lactic acid. Additionally, LDHA expression is stronger in GBMs than in other grades of gliomas. This is consistent with the CSF lactic acid level. Thus, the CSF lactic acid level appears to reflect the metabolic condition of the glioma tissues.

The CSF levels of citric and isocitric acid, which are TCA cycle metabolites, were significantly elevated in GBMs compared with grades I–II or III gliomas. Citric acid accumulates in tissue in which the glycolytic rate exceeds the TCA cycle activity [40]. Furthermore, it is well known that citric acid is required to produce cytoplasmic acetyl-CoA for lipid synthesis, and this step is essential to support cell growth [31, 41]. In an MRS study of gliomas, citric acid levels were reported to be high in pediatric pontine gliomas [42]. In addition, citric acid levels were higher in aggressive pediatric astrocytomas than in indolent astrocytomas [43]. Based on this evidence, it appears that high-grade gliomas have higher levels of citric acid than low-grade gliomas. In the present study, the higher CSF level of citric acid tended to be associated with a shorter OS in all glioma patients. Thus, measuring the amount of CSF citric acid may be useful for predicting the prognosis of gliomas.

*IDH* mutations were frequently observed in astrocytic and oligodendroglial tumors of grades II and III [14]. In the present study, the differences in the metabolic profiles by the *IDH* status were analyzed in grades I–III gliomas, excluding GBMs, because all GBMs did not have a *IDH* mutation and because several metabolites were significantly different from other grades of gliomas. Reitman et al. [44] reported that amino acid, choline lipid, and TCA cycle metabolite levels were altered in cells expressing IDH mutants. They described that the late TCA intermediates fumarate and malate were reduced in IDH mutant-expressing cells. Additionally, Lazovic et al. [45] recently reported that the ratio of lactate/choline was significantly increased in IDH1R132H-transfected U87 cells compared with control cells in an MRS study. These results are consistent with our results. Metabolic changes by *IDH* mutations are reported to be different among cell types [46, 47]; however, further studies are necessary. We discovered that CSF concentrations of pyruvate + oxaloacetic acid were significantly lower in the gliomas with *IDH* mutations compared with those with a wild-type *IDH*. In contrast, the CSF concentrations of lactic, citric, and isocitric acid were significantly higher in gliomas with *IDH* mutations compared with those of a wild-type *IDH*. In addition, the levels of the late TCA cycle metabolites that act downstream of isocitric acid decreased in the gliomas with *IDH* mutations. IDH1 converts isocitrate into α-ketoglutarate, which may explain the observed lower levels of late TCA metabolites (which are downstream from α-ketoglutarate) and increased isocitrate, pyruvate, and/or citrate levels (which are upstream from isocitrate). From these data, CSF metabolite levels may reflect the metabolic changes caused by the *IDH* mutations in the glioma cells. Recently, biochemical studies revealed that mutant IDH1 protein gains the function to catalyze the reduction of α-ketoglutarate to 2-hydroxyglutarate (2-HG) in a NADPH-consuming manner [48]. Sahm et al. [49] reported the success of the detection of 2-HG in glioma tissue using GC/MS. However, our study, which used the GCMS-QP2010 Ultra and Plus, did not detect 2-HG in the CSF of the glioma patients with the *IDH* mutation. Further studies are required to detect 2-HG in the CSF of glioma patients. Most recently, Locasale et al. [50] analyzed the CSF metabolic profiles from the 10 patients with malignant gliomas using LC–MS/MS and identified 124 polar metabolites. They also identified significant differences in the CSF metabolite composition between patients with malignant gliomas and controls. Thus, they described that the CSF metabolite composition may provide clinically relevant biomarkers and insights into the mechanisms underlying the pathogenesis of malignant gliomas.

The present study has several limitations. First, there are several histological types in the same WHO grade. The low-grade gliomas in this series contained four different histological types (i.e., diffuse astrocytoma, oligodendroglioma, ependymoma and pilocytic astrocytoma), and the grade III gliomas contained three different histological diagnoses (i.e., anaplastic astrocytoma, anaplastic oligodendroglioma, and anaplastic ependymoma). There may be significant differences in the metabolic profile among different histological types of gliomas. Therefore, the differences in the metabolite profiles of different histological types could have contributed to the differences observed among the different grades. Second, the relatively small sample size limited our power to detect potentially important associations between CSF metabolites and the clinical outcomes of interest. Despite these and other limitations, this is the first comparative report of the CSF metabolite profile among the various grades of gliomas. However, further studies will be needed to determine the CSF metabolome of various histological types of gliomas.

In conclusion, the CSF levels of several metabolites, such as citric, isocitric, and lactic acid, were altered between glioma grades and the IDH mutation status, which may reflect the glioma cell metabolism. Our study indicates that an analysis of the CSF metabolite levels may be useful for predicting glioma malignancy and prognosis.

## Electronic supplementary material

Below is the link to the electronic supplementary material.

Supplementary material 1 (DOC 168 kb)

Supplementary Figure S1 MRI scans of the gliomas. The MRI shows an enhanced tumor proximal to the ventricles (a, b) and distal to the ventricles (c, d). (TIFF 430 kb)

Supplementary Figure S2 Significant differences of the CSF metabolites related to TCA cycle and glycolisis between anaplastic astrocytomas (AAs) and GBMs. The columns are the average of each group; bars, SD. *p* values were calculated using the Mann–Whitney nonparametric *U* test (*p < 0.05). Q-value of citric acid, isocitric acid, and fumaric acid are 0.0039, 0.0039, and 0.0706, respectively. (TIFF 67 kb)

Supplementary Figure S3 (a-g) LDHA expression in various histological types of gliomas. a: pilocytic astrocytoma (patient-1), b: diffuse astrocytoma (patient-5), c: oligodendroglioma (patient-7), d: anaplastic astrocytoma (patient-13), e: anaplastic oligodendroglioma (patient-16), f: anaplastic ependymoma (patient-18), g: GBM (patient-26). (h,i) LDHA expression in GBM. h: LDHA is predominantly expressed in tumor cells rather than in vascular tissues. i: tumor cells near necrotic tissues markedly expresses LDHA. (Original magnification: a-h: ×200. i-j: ×100, k-l: ×200). (TIFF 869 kb)

Supplementary Figure S4 MRI (left panels) and 1H-MRS (right panels) findings for three glioma patients. Upper panels (a, b) Ependymoma (grade II) of 25-year old man (patient 10). (a) Fluid attenuated inversion recovery (FLAIR) MR image shows the tumor located in left lateral ventricle. (b) The MR spectra show that the lactic acid signal is barely detectable. In this patient, the relative value of CSF lactic acid measured by a GCMS-QP2010 Ultra is 2.13. Middle panels (c, d) Glioblastoma (grade IV) of 39-year old woman (patient 19). (c) FLAIR MRI shows the large tumor diffusely extended into the bilateral frontal lobes and corpus callosum. (d) In the MR spectra, lactic acid is detectable, but the peak is small. In this patient, the relative value of CSF lactic acid is 2.68. Lower panels (e, f) Glioblastoma (grade IV) of 54-year old woman (patient 21). (e) Gd-enhanced MRI shows heterogeneously Gd-enhanced tumor in the white matter of the left parietal lobe. (f) In the MR spectra, the peak of lactic acid is large. The relative value of CSF lactic acid is 3.07. (TIFF 325 kb)

Supplementary Figure S5 A: In survival analysis using the Kaplan–Meier method for all glioma patients, higher age (>50) were significantly associated with having a shorter OS compared with lower age (<50) (log-rank *p* = 0.004, HR = 7.81, 95 %CI: 1.53–39.8). B: High-grade gliomas (Grade III & IV) have a tendency of a shorter OS as compared with low-grade gliomas (grade I & II) (log-rank *p* = 0.057, HR = 5.8, 95 %CI: 0.734–45.91). C: Survival analysis of each grades of gliomas. GBMs have a tendency of a shorter OS compared with other grades of gliomas. (TIFF 74 kb)
