# Supplementary material for: GC/MS-based metabolomic analysis of cerebrospinal fluid (CSF) from glioma patients
Source: J Neurooncol. 2013 Mar 1;113(1):65–74. doi: 10.1007/s11060-013-1090-x (PMC3637650; doi:10.1007/s11060-013-1090-x)
Supplement: Supplementary file 1 — Supplementary material 1 (DOC 168 kb) [file 11060_2013_1090_MOESM1_ESM.doc]

Supplementary Table S1.

A comparison of the metabolite levels identified by GCMS-QP2010 Plus in grades I-II, grade III glioma, and GBM patients.

| Compound  name | Fold induction | | |
| --- | --- | --- | --- |
|  | GradeIII / Grades I-II | GBM / Grades I-II | GBM / Grade III |
| Succinic acid | 1.55 | 0.90 | 0.58 |
| Fumaric acid | 1.65 | 0.74 | 0.45 |
| Malic acid | 2.94 | 0.19 | 0.066 |
| Aconitic acid | 0.90 | 1.55 | 1.72 |
| Isocitric acid | 0.98 | **1.78**** | **1.83*** |
| Citric acid | 0.97 | **1.77*** | **1.83*** |
| Alanine | 0.83 | 1.05 | 1.27 |
| Valine | 0.76 | 1.06 | 1.40 |
| Leucine | 0.82 | 1.06 | 1.29 |
| Isoleucine | 0.78 | 0.99 | 1.26 |
| Proline | 0.52 | 0.60 | 1.15 |
| Serine | 1.01 | 0.82 | 0.81 |
| Threonine | 0.87 | 1.00 | 1.16 |
| Methionine | 0.93 | 1.02 | 1.10 |
| Phenylalanine | 0.93 | 0.12 | 0.090 |
| Tyrosine | 1.01 | 1.04 | 1.03 |

The values represent the fold induction of the peak intensity value. The peak intensity value was calculated for each quantified ion and normalized to that of 2-isopropylmalic acid as an internal standard. *p* values were calculated using the Steel-Dwass test. (* *p*<0.05, ** *p*<0.01)

Supplementary Table S2. List of CSF metabolites identified by a GCMS-QP2010 Ultra.

| No. | Retention time | *m/z* | Compound name |
| --- | --- | --- | --- |
|  | (min) | quantified |  |
| 1 | 4.101 | 148 | Propyleneglycol |
| 2 | 4.693 | 89 | Pyruvate+Oxaloacetic acid |
| 3 | 4.796 | 147 | Lactic acid |
| 4 | 4.997 | 147 | Glycolic acid |
| 5 | 5.563 | 205 | Hydroxybutyrate |
| 6 | 5.941 | 147 | 3-Hydroxy-butyrate |
| 7 | 6.832 | 147 | Urea |
| 8 | 6.974 | 105 | Benzoic acid |
| 9 | 7.08 | 201 | n-Caprylic acid |
| 10 | 7.088 | 299 | Phosphate |
| 11 | 7.089 | 205 | Glycerol |
| 12 | 7.475 | 86 | Glycine (3TMS) |
| 13 | 7.649 | 147 | Glyceric acid |
| 14 | 8.014 | 117 | Nonanoic acid |
| 15 | 9.144 | 217 | Threitol |
| 16 | 9.209 | 217 | meso-Erythritol |
| 17 | 9.469 | 230 | Pyroglutamic acid |
| 18 | 9.721 | 115 | Creatinine |
| 19 | 10.458 | 217 | Arabinose |
| 20 | 10.504 | 132 | Lauric acid |
| 21 | 10.891 | 147 | Arabitol |
| 22 | 10.933 | 103 | Ribitol |
| 23 | 11.373 | 156 | Glutamine |
| 24 | 11.647 | 274 | 2-Aminopimelic acid |
| 25 | 11.673 | 174 | 1-Methylhistidine |
| 26 | 11.827 | 103 | Tagatose_1 |
| 27 | 11.874 | 217 | Psicose_1 |
| 28 | 11.914 | 147 | 1,5-Anhydro-D-glucitol |
| 29 | 11.953 | 103 | Tagatose_2 |
| 30 | 11.988 | 217 | a-Sorbopyranose_1 |
| 31 | 12.016 | 217 | a-Sorbopyranose_2 |
| 32 | 12.05 | 148 | Allose_1 |
| 33 | 12.052 | 103 | Fructose_2 |
| 34 | 12.091 | 157 | 2-Dehydro-D-gluconate_1 |
| 35 | 12.092 | 147 | Mannose_1 |
| 36 | 12.129 | 205 | Galactose_1 |
| 37 | 12.177 | 205 | Glucose_1 |
| 38 | 12.218 | 103 | Mannose_2 |
| 39 | 12.321 | 147 | Glucose_2 |
| 40 | 12.396 | 319 | Mannitol |
| 41 | 12.397 | 217 | Glulcono-1,4-lactone |
| 42 | 12.405 | 317 | Lysine (4TMS) |
| 43 | 12.438 | 205 | Galactosamine_1 |
| 44 | 12.441 | 333 | Glucuronate_1 |
| 45 | 12.467 | 217 | Galactitol |
| 46 | 12.471 | 117 | Glucosamine_2 |
| 47 | 13.486 | 217 | Inositol |
| 48 | 13.545 | 441 | Uric acid |
| 49 | 15.838 | 217 | Inosine |

TMS: trimethylsilyl.

Supplementary Table S3.

A comparison of the metabolite levels identified by a GCMS-QP2010 Ultra in grades I-II, grade III glioma, and GBM patients. The metabolites that produced *p* values<0.05 or the metabolites involved in the TCA cycle and glycolysis are listed.

| Compound Name | Fold induction | | |
| --- | --- | --- | --- |
|  | Grade III /  Grade I-II | GBM /  Grade I-II | GBM /  Grade III |
| Pyruvate+Oxaloacetic acid | 1.47 | 1.35 | 0.92 |
| Lactic acid | 1.03 | **1.21*** | 1.18 |
| Glucose_1 | 0.98 | 0.97 | 0.99 |
| Glucose_2 | 0.95 | 0.97 | 1.02 |
| 2-Aminopimelic acid | 1.18 | **1.87*** | 1.58 |

The values are the fold induction of the peak intensity value. The peak intensity value was calculated for each quantified ion and normalized to that of 2-isopropylmalic acid as an internal standard. *p* values were calculated using the Steel-Dwass test. (**p*<0.05)

Supplementary Table S4. False discovery rate (FDR) of each metabolites.

| **Targeted, quantitative analysis using GCMS-QP2010 Plus** | | | | | | |
| --- | --- | --- | --- | --- | --- | --- |
|  | grade I-II vs. III | | grade I-II vs. IV | | grade III vs. VI | |
|  | p-value | Q-value | p-value | Q-value | p-value | Q-value |
| Succinic acid | 0.9827 | 1.0000 | 0.8713 | 0.9995 | 0.7732 | 0.3169 |
| Fumaric acid | 0.4802 | 1.0000 | 0.9981 | 0.9995 | 0.4973 | 0.2982 |
| Malic acid | 0.8320 | 1.0000 | 0.9115 | 0.9995 | 0.8122 | 0.3169 |
| Aconitic acid | 0.9324 | 1.0000 | 0.1535 | 0.6140 | 0.0857 | 0.1190 |
| Isocitric acid | 0.9956 | 1.0000 | **0.0096** | **0.1002** | **0.0174** | **0.0522** |
| Citric acid | 1.0000 | 1.0000 | **0.0125** | **0.1002** | **0.0174** | **0.0522** |
| Alanine | 0.9730 | 1.0000 | 0.2402 | 0.7687 | 0.0928 | 0.1190 |
| Valine | 0.6660 | 1.0000 | 0.7422 | 0.9995 | 0.1980 | 0.1815 |
| Leucine | 0.4780 | 1.0000 | 0.9337 | 0.9995 | 0.2422 | 0.1815 |
| Isoluecine | 0.5899 | 1.0000 | 0.9702 | 0.9995 | 0.4764 | 0.2982 |
| Proline | 0.5067 | 1.0000 | 0.8118 | 0.9995 | 0.8122 | 0.3169 |
| Serine | 0.9149 | 1.0000 | 0.9995 | 0.9995 | 0.6680 | 0.3169 |
| Threonine | 0.9318 | 1.0000 | 0.6704 | 0.9995 | 0.2260 | 0.1815 |
| Methionine | 0.9902 | 1.0000 | 0.7076 | 0.9995 | 0.5605 | 0.3056 |
| Phenylalanine | 0.9955 | 1.0000 | 0.1157 | 0.6140 | 0.0993 | 0.1190 |
| Tyrosine | 0.8525 | 1.0000 | 0.6107 | 0.9995 | 0.8455 | 0.3169 |

| **Non-targeted, semi-quantitative analysis by GCMS-QP2010 Ultra** | | | | | | |
| --- | --- | --- | --- | --- | --- | --- |
|  | grade I-II vs. III | | grade I-II vs. IV | | grade III vs. VI | |
|  | p-value | Q-value | p-value | Q-value | p-value | Q-value |
| Propyleneglycol | 0.9731 | 0.9989 | 0.9787 | 1.0000 | 0.9870 | 0.9967 |
| Pyruvate+Oxalacetic acid | 0.1773 | 0.9989 | 0.6464 | 1.0000 | 0.9967 | 0.9967 |
| Lactic acid | 0.7300 | 0.9989 | **0.0327** | 0.9554 | 0.0649 | 0.9967 |
| Glycolic acid | 0.7569 | 0.9989 | 0.9786 | 1.0000 | 0.5987 | 0.9967 |
| HydroxyButyrate | 0.8550 | 0.9989 | 0.2532 | 1.0000 | 0.7668 | 0.9967 |
| 3-Hydroxy-Butyrate | 0.9480 | 0.9989 | 0.9418 | 1.0000 | 0.9489 | 0.9967 |
| Urea | 0.3297 | 0.9989 | 0.7084 | 1.0000 | 0.3887 | 0.9967 |
| Benzoic acid | 0.8969 | 0.9989 | 0.9418 | 1.0000 | 0.9489 | 0.9967 |
| n-Caprylic acid | 0.9327 | 0.9989 | 0.5421 | 1.0000 | 0.9606 | 0.9967 |
| Phosphate | 0.8969 | 0.9989 | 0.8579 | 1.0000 | 0.4328 | 0.9967 |
| Glycerol | 0.9957 | 0.9989 | 0.4221 | 1.0000 | 0.5264 | 0.9967 |
| Glycine(3TMS) | 0.4273 | 0.9989 | 0.2986 | 1.0000 | 0.9489 | 0.9967 |
| Glyceric acid | 0.3765 | 0.9989 | 0.9976 | 1.0000 | 0.3887 | 0.9967 |
| Nonanoic acid(C9) | 0.9989 | 0.9989 | 0.9976 | 1.0000 | 0.9489 | 0.9967 |
| Threitol | 0.3297 | 0.9989 | 0.8892 | 1.0000 | 0.5750 | 0.9967 |
| meso-erythritol | 0.9957 | 0.9989 | 0.3489 | 1.0000 | 0.7205 | 0.9967 |
| Pyroglutamic acid | 0.9327 | 0.9989 | 0.1997 | 1.0000 | 0.7205 | 0.9967 |
| Creatinine | 0.8081 | 0.9989 | 0.8579 | 1.0000 | 0.6727 | 0.9967 |
| Arabinose | 0.8081 | 0.9989 | 0.9905 | 1.0000 | 0.9489 | 0.9967 |
| Lauric acid | 0.4531 | 0.9989 | 0.3847 | 1.0000 | 0.9357 | 0.9967 |
| Arabitol | 0.3769 | 0.9989 | 0.6673 | 1.0000 | 0.8107 | 0.9967 |
| Ribitol | 0.9616 | 0.9989 | 0.7485 | 1.0000 | 0.6727 | 0.9967 |
| Glutamine | 0.9327 | 0.9989 | 0.4221 | 1.0000 | 0.4789 | 0.9967 |
| 2-Aminopimelic acid | 0.7032 | 0.9989 | **0.0425** | 0.9554 | 0.2371 | 0.9967 |
| 1-Methyl Histidine | 0.9957 | 0.9989 | 0.5011 | 1.0000 | 0.5750 | 0.9967 |
| Tagatose_1 | 0.2097 | 0.9989 | 0.7870 | 1.0000 | 0.0933 | 0.9967 |
| Psicose_1 | 0.5909 | 0.9989 | 0.6877 | 1.0000 | 0.3887 | 0.9967 |
| 1,5-Anhydro-D-glucitol | 0.9327 | 0.9989 | 0.5421 | 1.0000 | 0.3072 | 0.9967 |
| a-Sorbopyranose_1 | 0.8969 | 0.9989 | 0.5011 | 1.0000 | 0.2708 | 0.9967 |
| Allose_1 | 0.9827 | 0.9989 | 0.5009 | 1.0000 | 0.2708 | 0.9967 |
| Fructose_2 | 0.9616 | 0.9989 | 0.4221 | 1.0000 | 0.2371 | 0.9967 |
| 2-Dehydro-D-gluconate_1 | 0.9327 | 0.9989 | 0.6255 | 1.0000 | 0.5264 | 0.9967 |
| Mannose_1 | 0.9615 | 0.9989 | 0.6045 | 1.0000 | 0.5259 | 0.9967 |
| Galactose_1 | 0.6476 | 0.9989 | 0.6256 | 1.0000 | 0.3468 | 0.9967 |
| Glucose_1 | 0.8079 | 0.9989 | 0.9905 | 1.0000 | 0.8885 | 0.9967 |
| Mannitol | 0.5351 | 0.9989 | 1.0000 | 1.0000 | 0.3074 | 0.9967 |
| Gulcono-1,4-lactone | 0.5912 | 0.9989 | 0.9905 | 1.0000 | 0.4328 | 0.9967 |
| Lysine(4TMS) | 0.8550 | 0.9989 | 0.9905 | 1.0000 | 0.8515 | 0.9967 |
| Galactosamine_1 | 0.8081 | 0.9989 | 0.5421 | 1.0000 | 0.1782 | 0.9967 |
| Glucuronate_1 | 0.8081 | 0.9989 | 0.9300 | 1.0000 | 0.2708 | 0.9967 |
| Galactitol | 0.7032 | 0.9989 | 0.6878 | 1.0000 | 0.9055 | 0.9967 |
| Glucosamine_2 | 0.9957 | 0.9989 | 1.0000 | 1.0000 | 0.8515 | 0.9967 |
| Inositol | 0.9616 | 0.9989 | 0.3664 | 1.0000 | 0.9213 | 0.9967 |
| Uric acid | 0.9616 | 0.9989 | 0.8579 | 1.0000 | 0.8107 | 0.9967 |
| Inosine | 0.9827 | 0.9989 | 0.5421 | 1.0000 | 0.4789 | 0.9967 |

| Anaplastic astrocytoma (grade III) vs. GBM (grade IV) | | |
| --- | --- | --- |
|  | p-value | Q-value |
| Citric acid | **0.01242935** | **0.03982533** |
| Isocitric acid | **0.01242935** | **0.03982533** |
| Fumaric acid | **0.03306801** | **0.07063626** |
| Succinic acid | 0.3303582 | 0.161314 |
| Malic acid | 0.2663654 | 0.1422451 |
| Aconitic acid | 0.1148732 | 0.09994707 |
| L-Leucine | 0.09445445 | 0.09994707 |
| L-Isoluecine | 0.1947286 | 0.1247874 |
| L-Proline | 0.458902 | 0.1960512 |
| L-Serine | 0.3524189 | 0.161314 |
| L-Threonine | 0.1247726 | 0.09994707 |
| L-Methionine | 0.2657349 | 0.1422451 |
| L-Phenylalanine | 0.06058727 | 0.07765192 |
| L-Tyrosine | 0.9627337 | 0.3855912 |
| L-Alanine | 0.04595873 | 0.07362899 |
| L-Valine | 0.1630576 | 0.1161019 |

| **IDH mutation (+) vs. IDH mutation (-)** | | |
| --- | --- | --- |
|  | p-value | Q-value |
| Succinic acid | 1.0000 | 0.8612 |
| Fumaric acid | 0.1898 | 0.2551 |
| Malic acid | 0.5425 | 0.5339 |
| Aconitic acid | 0.2222 | 0.2551 |
| Isocitric acid | **0.0130** | **0.0447** |
| Citric acid | **0.0114** | **0.0447** |
| Lactic acid | **0.0312** | **0.0717** |
| Pyruvate+Oxaloacetic acid | **0.0492** | **0.0847** |
