# Supplementary figures and images for: GC/MS-based metabolomic analysis of cerebrospinal fluid (CSF) from glioma patients
Source: J Neurooncol. 2013 Mar 1;113(1):65–74. doi: 10.1007/s11060-013-1090-x (PMC3637650; doi:10.1007/s11060-013-1090-x)

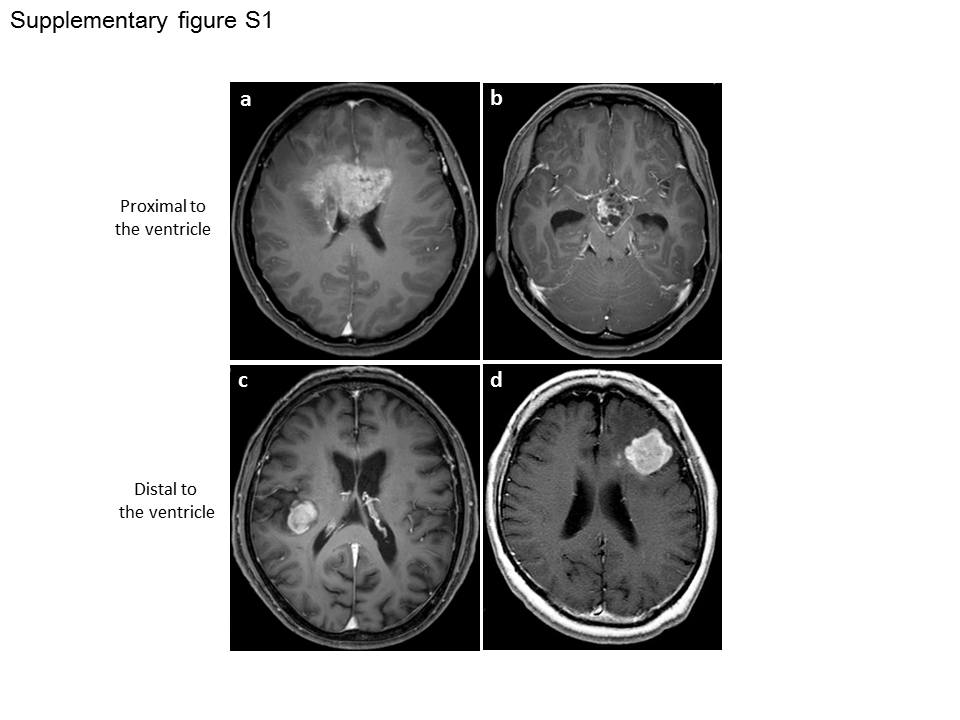

Supplement: Supplementary file 2 — MRI scans of the gliomas. The MRI shows an enhanced tumor proximal to the ventricles (a, b) and distal to the ventricles (c, d). (TIFF 430 kb) [file 11060_2013_1090_MOESM2_ESM.tif]

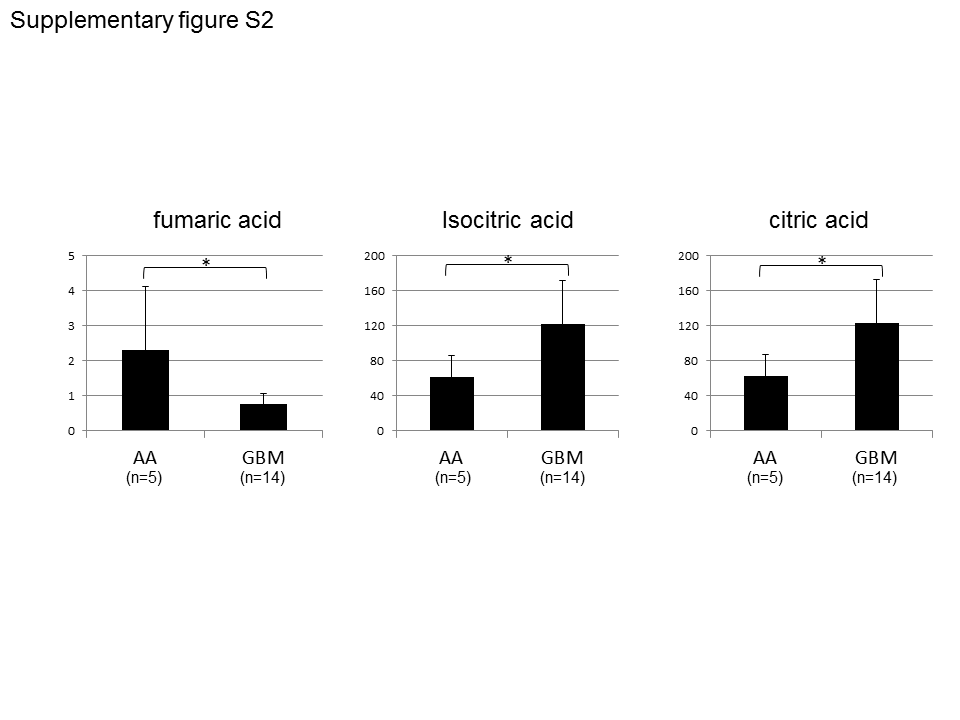

Supplement: Supplementary file 3 — Significant differences of the CSF metabolites related to TCA cycle and glycolisis between anaplastic astrocytomas (AAs) and GBMs. The columns are the average of each group; bars, SD. p values were calculated using the Mann–Whitney nonparametric U test (*p < 0.05). Q-value of citric acid, isocitric acid, and fumaric acid are 0.0039, 0.0039, and 0.0706, respectively. (TIFF 67 kb) [file 11060_2013_1090_MOESM3_ESM.tif]

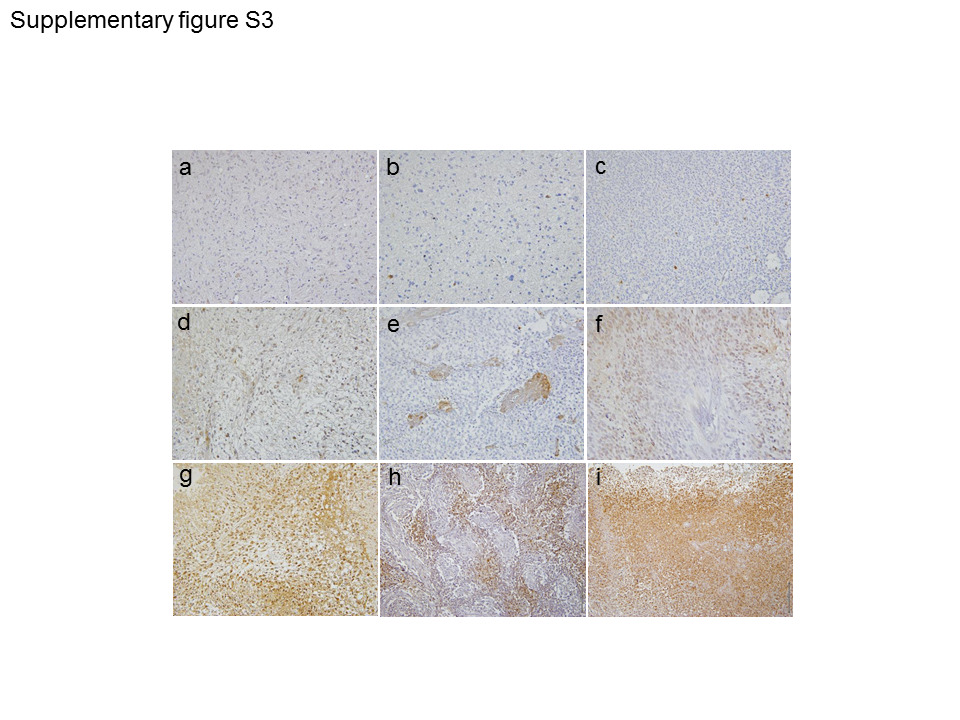

Supplement: Supplementary file 4 — (a-g) LDHA expression in various histological types of gliomas. a: pilocytic astrocytoma (patient-1), b: diffuse astrocytoma (patient-5), c: oligodendroglioma (patient-7), d: anaplastic astrocytoma (patient-13), e: anaplastic oligodendroglioma (patient-16), f: anaplastic ependymoma (patient-18), g: GBM (patient-26). (h,i) LDHA expression in GBM. h: LDHA is predominantly expressed in tumor cells rather than in vascular tissues. i: tumor cells near necrotic tissues markedly expresses LDHA. (Original magnification: a-h: ×200. i-j: ×100, k-l: ×200). (TIFF 869 kb) [file 11060_2013_1090_MOESM4_ESM.tif]

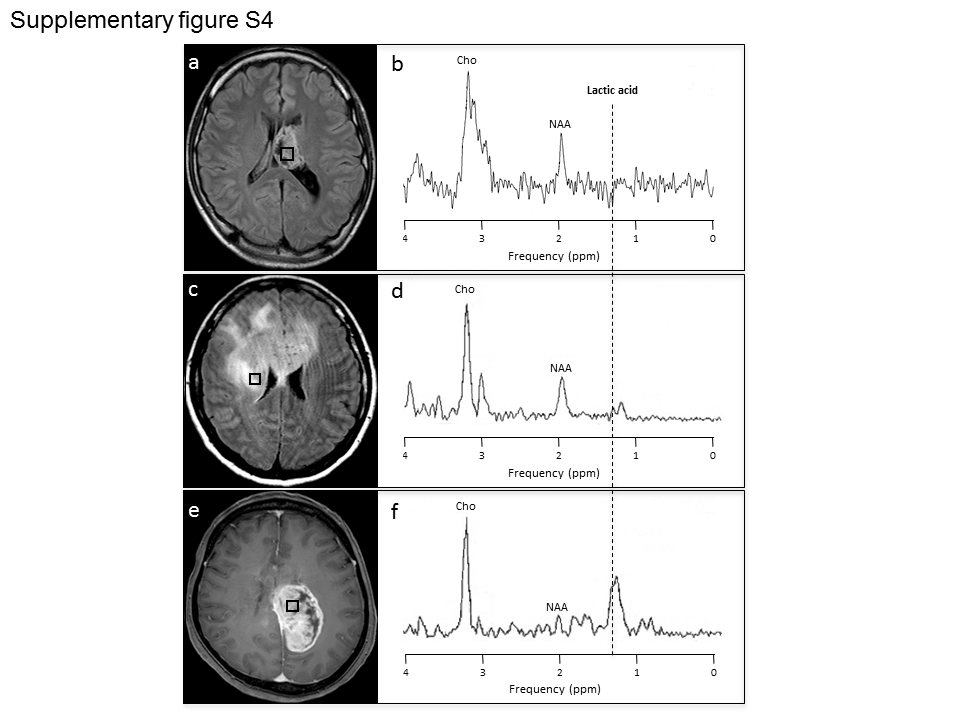

Supplement: Supplementary file 5 — MRI (left panels) and 1H-MRS (right panels) findings for three glioma patients. Upper panels (a, b) Ependymoma (grade II) of 25-year old man (patient 10). (a) Fluid attenuated inversion recovery (FLAIR) MR image shows the tumor located in left lateral ventricle. (b) The MR spectra show that the lactic acid signal is barely detectable. In this patient, the relative value of CSF lactic acid measured by a GCMS-QP2010 Ultra is 2.13. Middle panels (c, d) Glioblastoma (grade IV) of 39-year old woman (patient 19). (c) FLAIR MRI shows the large tumor diffusely extended into the bilateral frontal lobes and corpus callosum. (d) In the MR spectra, lactic acid is detectable, but the peak is small. In this patient, the relative value of CSF lactic acid is 2.68. Lower panels (e, f) Glioblastoma (grade IV) of 54-year old woman (patient 21). (e) Gd-enhanced MRI shows heterogeneously Gd-enhanced tumor in the white matter of the left parietal lobe. (f) In the MR spectra, the peak of lactic acid is large. The relative value of CSF lactic acid is 3.07. (TIFF 325 kb) [file 11060_2013_1090_MOESM5_ESM.tif]

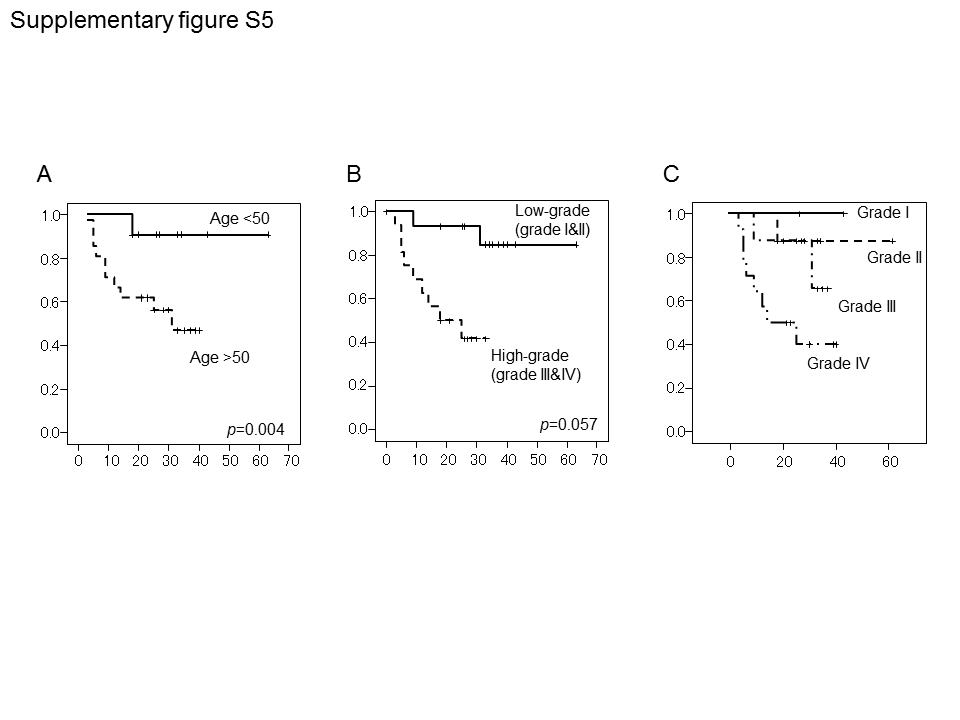

Supplement: Supplementary file 6 — A: In survival analysis using the Kaplan–Meier method for all glioma patients, higher age (>50) were significantly associated with having a shorter OS compared with lower age (<50) (log-rank p = 0.004, HR = 7.81, 95 %CI: 1.53–39.8). B: High-grade gliomas (Grade III & IV) have a tendency of a shorter OS as compared with low-grade gliomas (grade I & II) (log-rank p = 0.057, HR = 5.8, 95 %CI: 0.734–45.91). C: Survival analysis of each grades of gliomas. GBMs have a tendency of a shorter OS compared with other grades of gliomas. (TIFF 74 kb) [file 11060_2013_1090_MOESM6_ESM.tif]
